# Reverse Epidemiology: An Experimental Framework to Drive *Leishmania* Biomarker Discovery *in situ* by Functional Genetic Screening Using Relevant Animal Models

**DOI:** 10.3389/fcimb.2018.00325

**Published:** 2018-09-19

**Authors:** Laura Piel, Pascale Pescher, Gerald F. Späth

**Affiliations:** ^1^Institut Pasteur, Unité de Parasitologie Moléculaire et Signalisation, INSERM U1201, Paris, France; ^2^Université Paris Diderot, Sorbonne Paris Cité, Paris, France

**Keywords:** *Leishmania*, biomarker discovery, reverse epidemiology, cosmid screen, functional genetics

## Abstract

*Leishmania* biomarker discovery remains an important challenge that needs to be revisited in light of our increasing knowledge on parasite-specific biology, notably its genome instability. In the absence of classical transcriptional regulation in these early-branching eukaryotes, fluctuations in transcript abundance can be generated by gene and chromosome amplifications, which have been linked to parasite phenotypic variability with respect to virulence, tissue tropism, and drug resistance. Conducting *in vitro* evolutionary experiments to study mechanisms of *Leishmania* environmental adaptation, we recently validated the link between parasite genetic amplification and fitness gain, thus defining gene and chromosome copy number variations (CNVs) as important *Leishmania* biomarkers. These experiments also demonstrated that long-term *Leishmania* culture adaptation can strongly interfere with epidemiologically relevant, genetic signals, which challenges current protocols for biomarker discovery, all of which rely on *in vitro* expansion of clinical isolates. Here we propose an experimental framework independent of long-term culture termed “reverse” epidemiology, which applies established protocols for functional genetic screening of cosmid-transfected parasites in animal models for the identification of clinically relevant genetic loci that then inform targeted field studies for their validation as *Leishmania* biomarkers.

## Introduction

Biomarkers are defined as biological characteristics that are objective and quantifiable indicators for responses to therapeutic interventions, or normal and pathogenic biological processes (Biomarkers Definitions Working Group 2001, 2001). With respect to *Leishmania* infection, we can distinguish direct biomarkers that are applied to determine parasite species and prevalence (e.g., parasite-specific proteins, lipids, transcripts, genetic loci), and indirect biomarkers that correspond to different correlates of the host anti-microbial response [e.g., adenosine deaminase (ADA) or cytokines such as IL-10 or TNF] (Kip et al., 2015).

Direct *Leishmania* biomarkers can have either purely diagnostic value (e.g., kinetoplast (k) DNA, ribosomal small sub-unit (SSU) RNA, HSP70 locus, carbohydrate antigens), or prognostic value allowing for the prediction of treatment outcome or disease evolution (e.g., dissemination in cutaneaous leishmaniasis or development of post-kala-azar dermal leishmaniasis in visceral leishmaniasis). However, despite their potentially important impact on clinical management of leishmaniasis, only few biomarker candidates with potential prognostic value are described, most of which are linked to drug resistance (Vanaerschot et al., 2012; Torres et al., 2013; Hefnawy et al., 2017; Ponte-Sucre et al., 2017). The absence of this class of markers is explained by various biological and technical constraints, some of which are linked to *Leishmania* genome instability that limits biomarker discovery and needs to be considered in ongoing and future biomarkers discovery campaigns.

In the absence of classical transcriptional regulation, *Leishmania* often regulates transcript and protein abundance by chromosome and gene copy number variations (CNVs) (Dumetz et al., 2017; Prieto Barja et al., 2017), which can drive environmental adaptation (Leprohon et al., 2009; Downing et al., 2011; Rogers et al., 2011; Brotherton et al., 2013; Mukherjee et al., 2013; Ubeda et al., 2014; Zhang et al., 2014; Laffitte et al., 2016). Our recent demonstration that karyotypic fluctuations and haplotype selection allow for fitness gain in culture reveals the importance of *Leishmania* genome plasticity in short-term evolutionary adaptation (Prieto Barja et al., 2017). Conceivably, the highly dynamic genomic changes occurring during culture adaptation challenge past and current protocols in *Leishmania* biomarker discovery, which rely on adaptation and mass-expansion of field isolates in culture prior to analysis, often resulting in loss of epidemiologically relevant, genetic signals. Here, by drawing from the current literature, we propose an alternative strategy independent of long-term culture that is based on functional genetic screening in relevant animal models. Our review provides an overview on past functional screening results and their documented success in revealing genomic loci that are under environmental selection, and advocates for *Leishmania* biomarker discovery by combining cosmid selection and subsequent clinical validation, an experimental framework we termed “reverse” epidemiology. In the following we summarize studies that developed and applied cosmid-based approaches to identify new *Leishmania* factors linked to parasite pathogenicity, tropism and drug resistance, and discuss the potential epidemiological relevance of these factors where clinical data were available.

## Cosmid-based functional genetic screening in *Leishmania*

Various genetic methods have been successfully applied in the past to identify *Leishmania* genes or genetic markers that are associated with disease outcome or clinical manifestation, including whole genome sequencing (WGS) of isolates (Downing et al., 2011; Rogers et al., 2011; Leprohon et al., 2015), random amplification of polymorphic DNA (RAPD) (Bhattacharyya et al., 1993; Schönian et al., 1996; Mkada-Driss et al., 2014), or assessment of amplified fragment length polymorphisms (AFLP) (Kumar et al., 2009, 2010a; Odiwuor et al., 2011; Jaber et al., 2018). Likewise, cosmid-based functional screens have been applied to discover clinically relevant loci. This approach is based on the genetic transfer of a given cellular phenotype (e.g., drug resistance) from a donor strain to a recipient strain via transfection of a cosmid library. While currently established WGS protocols for *Leishmania* biomarker discovery have been applied on clinical isolates maintained in long-term culture, causing potentially important bias, cosmid-based approaches can directly reveal clinically relevant genotype-phenotype relationships, especially when applied *in situ* in infected animals. Even though this functional genetic approach represents a powerful tool, this technology has not been applied in a systematic way at larger scale to drive biomarker discovery.

The preparation and application of a cosmid library is a complex procedure, where genomic DNA fragments of an appropriate size are cloned into purified cosmid DNA and packaged into phages for efficient bacterial transduction, which allows for amplification of the library and assessment of its genomic coverage prior to transfection into parasites by electroporation. The generation of a first series of *Leishmania* shuttle cosmid vectors and the validation of a protocol that allows for genetic complementation and functional screening in these parasites using genomic cosmid libraires was established in 1993 by Beverley and collaborators (Ryan et al., 1993a) followed by Kelly and collaborators in 1994 (Kelly et al., 1994). Subsequently, this protocol was applied in various studies for the identification of *Leishmania* pathogenicity and drug resistance genes.

### Cosmid-based identification of novel *Leishmania* pathogenicity factors

Key for *Leishmania* infectivity is the capacity of procyclic promastigotes to undergo differentiation into infectious metacyclic promastigotes able to resist to complement lysis encountered inside the mammal host following parasite transmission (Sacks and Perkins, 1984; Franke et al., 1985; Howard et al., 1987). Parasite resistance has been largely attributed to the surface glycolipid lipophosphoglycan (LPG), a major *Leishmania* virulence factor essential for *L. major* promastigote virulence (Späth et al., 2000, 2003a), that undergoes important modifications during metacyclogenesis (Sacks et al., 1990; Mcconville et al., 1992; Sacks, 2001). LPG biosynthetic genes and their virulence functions have been genetically identified combining cosmid screens with functional null mutant analysis and virulence assessment in macrophages and mice. LPG deficient mutants were generated by chemical mutagenesis, isolated by their failure to agglutinate in the presence of lectin (King and Turco, 1988), transfected with a cosmid library prepared from *L. donovani*, and screened for restoration of LPG expression using either lectin- or antibody-based agglutination assays revealing the two first LPG biosynthetic genes, a galactofuranose transferase encoded by the gene *lpg1* (Ryan et al., 1993b), and an UdP galactose transporter encoded by *lpg2* (Descoteaux et al., 1995). The virulence functions of both genes were confirmed in subsequent studies in *L. major lpg1* and *lpg2* null mutants (Späth et al., 2000, 2003b). Few years later, by combining cosmid library transfection and antibody panning, Dobson et al. identifed genes encoding arabinosyl- and galactosyltransferases that mediate developmental modifications of LPG during metacyclogenesis (Dobson et al., 2003a,b).

Cosmid-based functional screening has also been applied to gain insight into pathways that govern complement resistance in promastigotes revealing genes that are likely linked to metacyclogenesis. Based on the observation that decrease in resistance to complement lysis is a consequence of long-term maintenance in culture (Lincoln et al., 2004), Dahlin-Laborde et al. used genomic DNA from animal-derived *Leishmania infantum (chagasi)* promastigotes to construct a cosmid library that was transfected into long-term cultured parasites. The transfected parasites were subjected to complement lysis allowing for the selection of seven different cosmids that conferred increased complement resistance albeit at lower levels compared to short-term cultured control parasites. In-depth analysis of two cosmids revealed genomic fragments of *L. infantum* chromosome 36 (Dahlin-Laborde et al., 2005), with two sub-regions encoding, respectively, 5 and 13 genes shown to be critical for the phenotype, including an ADP-ribosylation factor-like protein and an ATP-dependent RNA helicase (Dahlin-Laborde et al., 2008). Cosmid screens were further applied by the Matlashewski team to identify virulence and visceralization factors using libraries prepared with genomic DNA from *L. donovani* transfected in *L. major* promastigotes. Transfectants expressing the heterologous library were inoculated into mice by tail vein or footpad injections and cosmids were recovered from parasites that established infection in spleen (type I), skin (type III), or both (type II). Subsequent analysis of individual ORFs by transgenic expression and infection validated an ORF encoding for an unknown protein and a 4.4 kb miniexon gene array on chromosome 36 (Zhang and Matlashewski, 2004). Unlike in *L. major*, overexpression of the miniexon region in *L. braziliensis* led to complete virulence attenuation in a hamster model (de Toledo et al., 2009), suggesting species-specific functions of this array. This is further supported by the genetic divergence of this array between new world and old world dermotropic species (Fernandes et al., 1994), which is used as a diagnostic signal for parasite genotyping (Serin et al., 2005; Ovalle-Bracho et al., 2016).

A final example documenting the power of cosmid-based approaches in identifying putative *Leishmania* virulence factors is represented by a complementation screen conducted using a cosmid library derived from an attenuated HSP100 null mutant that spontaneously recovered infectivity and/or pathogenicity in mice, likely by the amplification of a compensatory locus (Reiling et al., 2006). A screen conducted in mice using cosmid-transfected HSP100 null mutants and subsequent validation experiments revealed P46 as a new virulence factor (Reiling et al., 2010). A follow-up study by Bifeld et al applied a phylogenetic approach on 20 clinical isolates comparing P46 amino acid sequences thus establishing a strong correlation between P46 isoforms and their geographical origin. Transgenic parasites over-expressing three different P46 isoforms in a *L. major* lab strain were co-injected in BALB/c and C57BL/6 mice. Selection of different isoforms according to the mouse strain suggested that the P46 genetic polymorphism may be linked to parasite adaptation to genetically distinct, region-specific host reservoirs (Bifeld et al., 2015; Table 1).

**Table 1:** Genes identified by cosmid-based approach potentially linked to *Leishmania* pathogenicity.

**Gene**	**ID**	chr^*^	**Product**	**Function**	**Strain/Isolate**	**References**	Validation^**^
lpg1	LmjF.25.0010	25	Beta galactofuranosyl transferase	Galactofuranosyl transferase implicated in LPG biosynthesis	*L. donovani* L1S2D	Ryan et al., 1993b	Yes (Späth et al., 2000)
lpg2	LmjF.34.3120	34	Lipophosphoglycan biosynthetic protein 2	Transmembrane transporter activity	*L. donovani* 1S/Cl2D	Descoteaux et al., 1995	Yes (Späth et al., 2003b)
sca2	LmjF.02.0180	2	Phosphoglycan beta 1,2 arabinosyltransferase	Galactosyltransferase activity	*L. major* FV1	Dobson et al., 2003a	No
sca1	LmjF.02.0220	2	Phosphoglycan beta 1,2 arabinosyltransferase	Galactosyltransferase activity	*L. major* FV1	Dobson et al., 2003a	No
scg1	LmjF.07.1170	7	Phosphoglycan beta 1,3 galactosyltransferase 1	Galactosyltransferase activity	*L. major* FV1	Dobson et al., 2003b	No
scg2	LmjF.21.0010	21	Phosphoglycan beta 1,3 galactosyltransferase 2	Galactosyltransferase activity	*L. major* FV1	Dobson et al., 2003b	No
scg3	LmjF.02.0010	2	Phosphoglycan beta 1,3 galactosyltransferase 3	Galactosyltransferase activity	*L. major* FV1	Dobson et al., 2003b	No
scg4	LmjF.36.0010	36	phosphoglycan beta 1,3 galactosyltransferase 4	Galactosyltransferase activity	*L. major* FV1	Dobson et al., 2003b	No
scg5	LmjF.31.3190	31	phosphoglycan beta 1,3 galactosyltransferase 5	Galactosyltransferase activity	*L. major* FV1	Dobson et al., 2003b	No
scg6	LmjF.25.2460	25	Phosphoglycan beta 1,3 galactosyltransferase 6	Galactosyltransferase activity	*L. major* FV1	Dobson et al., 2003b	No
Miniexon		36	Miniexon		*L. donovani* 1S/Cl2D	Zhang and Matlashewski, 2004	No
P46	LmjF.33.3060	33	46 kD virulence factor	Unknown	*L. major* 5ASKH	Reiling et al., 2010	No
	LmJF36.0790 - LmjF.36.0840	36	Specific genes involved in phenotype not identified	Not applicable	*L. infantum* (*chagasi*)	Dahlin-Laborde et al., 2005	No
	LmJF36.0840 - LmjF.36.0900	36	Specific genes involved in phenotype not identified	Not applicable	*L. infantum* (Chagasi)	Dahlin-Laborde et al., 2008	No
	LmJF36.3090 - LmjF.36.3210	36	Specific genes involved in phenotype not identified	Not applicable	*L. infantum* (*chagasi*)	Dahlin-Laborde et al., 2008	No

* *chr, chromosome*;

** *validation refers to loss of function studies establishing a direct link between the gene and parasite pathogenicity*.

### Cosmid-based identification of *Leishmania* drug resistance genes

Since 1999, screening of cosmid libraries has been used as a gain-of-function strategy to identify drug resistance or drug tolerance genes (reviewed in Clos and Choudhury, 2006). Beverley and collaborators established the first proof-of-principle of this approach culturing cosmid transfected *L. major* parasites under pressure of the drugs methotrexate and tubercidin, which resulted in the selection of the known resistance genes *DHFR-TS, PTR1*, and *TOR* (Cotrim et al., 1999). The same study identified a new gene encoding a 63 kDa hypothetical protein located on chromosome 31 termed tubercidin-resistant protein (TRP) that is conserved in *Leishmania* and co-localizes in the endoplasmic reticulum in stationary phase promastigotes (Aoki et al., 2016).

Functional complementation has also been a powerful tool for the identification of transporters that can alter drug efficacy. The biopterin transporter bt1, previously named ORF G (Kundig et al., 1999), and the miltefosine (MIL) transporter LdMT (Perez-Victoria et al., 2003) were identified using *Leishmania tarentolae* transfected with a heterologous *L. mexicana* cosmid library selected under methotrexate pressure (showing that bt1 can confer resistance), and *L. donovani* MIL resistant parasites transfected with a *L. donovani* wild-type cosmid library subjected to MIL selection (showing that a non-mutated LdMT can restore susceptibility). Likewise, the cosmid approach was applied to screen for genes mediating resistance to two inhibitors of ergosterol biosynthesis, terbinafine, and itraconazole, which resulted in the selection of nine different cosmids, some of which conferred cross-resistance to both drugs, and the identification of squalene synthase 1 (SQS1) as an itraconazole resistance gene (Cotrim et al., 1999).

This approach has been recently applied to directly identify clinically relevant drug resistance loci by heterologous screening. Clos and collaborators prepared cosmid libraries from antimony SbIII/SbV resistant or SbIII sensitive/SbV resistant *L. braziliensis* field isolates that were transfected into SbIII sensitive/SbV resistant promastigotes. Culture under drug pressure selected for cosmids carrying a genomic fragment of chromosome 20, which also conferred drug resistance when transfected into *L. infantum* (Nühs et al., 2014). A competition assay with full-length or truncated derivatives of the cosmid insert validated ARM58 as a SbIII resistance gene. A more recent study performed by the same group with cosmid-transfected *L. infantum* extended this finding to the neighboring genes and defined a cluster of three genes, ARM58, ARM56 (previously named ARM58rel), and HSP23 at the telomere of the chromosome 34 that confer increased resistance of intracellular amastigotes against SbV (Tejera Nevado et al., 2016). Using a *L. infantum* cosmid library, the same team revealed a protein termed P299 that conferred increased resistance of intracellular amastigotes to MIL and reduced promastigote sensitivity to MIL and SbIII, but not pentamidin (Choudhury et al., 2008). Another gene—today annotated as cysteine leucine-rich protein (CLrP, LinJ.34.0570)—was revealed causing antimony resistance in *L. tarentolae* transfected with a cosmid library prepared from arsenite and SbIII resistant parasites (Brochu et al., 2004), and in *L. infantum* axenic amastigotes (Genest et al., 2008). Brochu et al. also reported members of the HSP70 protein family as important genes contributing to antimony tolerance, supporting recent phylogenetic evidence that HSP70 family members may allow parasite environmental adaptation with potential important consequences for drug susceptibility (Drini et al., 2016).

Recent work by the Ouellette team coupled cosmid selection and next generation sequencing for drug resistance and drug target gene discovery, proposing a high-throughput capable screening strategy the authors referred to as Cos-Seq (Gazanion et al., 2016). Screening cosmid transfected *L. infantum* against SbIII, amphotericin B, MIL, paramomycin or pentamidin revealed 64 enriched loci, including 12 common to at least two anti-leishmanial drugs, suggesting the existence of multi-drug resistance genes. This study validated 6 known and uncovered 7 new resistance genes in promastigotes, including two new genes causing methotrexate resistance both encoding for phosphatase 2C-like proteins (LinJ.34.2310 and LinJ.34.2320), one hypothetical protein with leucine-rich repeats causing both pentamidin and paromomycin resistance (LinJ.06.1010), a serine/threonine phosphatase causing SbIII resistance (LinJ.12.0610), and phospholipid-translocating ATPase (LinJ30.2270) and C-8 sterol isomerase (LinJ.29.2250) that were revealed screening for MIL resistance (Table 2).

**Table 2:** Genes identified by cosmid-based approach linked to *Leishmania* drug resistance or susceptibility.

**Gene**	**ID**	**Product**	**Function**	**Drug**	Strain^*^	Validation^**^	**References**
TRP	LmjF.31.2010	Turbicidin-resistant protein	ER protein	Tubercidin	*L. major*	No	Cotrim et al., 1999
bt1	LmxM.34.5150	Biopterin transporter	Folate/biopterin transport	Methotrexate	*L. mexicana*	No	Kundig et al., 1999
SQS1	LmjF.31.2940	Squalene synthase	Ergosterol biosynth	Itraconazole	*L. major*	No	Cotrim et al., 1999
ARM58	LbrM20.0210; LinJ.34.0220	58 kDa antimony resistance marker	Response to drug	SbIII	*L. braziliensis; L. infantum*	No	Nühs et al., 2014; Tejera Nevado et al., 2016
ARM56	LinJ.34.0210	56 kDa antimony resistance marker	Response to drug	SbIII	*L. braziliensis; L. infantum*	No	Nühs et al., 2014; Tejera Nevado et al., 2016
HSP23	LinJ.34.0230	HSP 23	Response to drug		*L. infantum*	No	Tejera Nevado et al., 2016
P299	LinJ.08.0630	P299	Response to drug	Miltefosine/ SbIII	*L. infantum*	Partially Downing et al., 2011; Jeddi et al., 2014	Choudhury et al., 2008
CLrP	LinJ.34.0570	Cysteine leucine rich protein	Response to drug	SbIII	*L. infantum*	Partially (Kumar et al., 2010b; Das et al., 2015)	Genest et al., 2008
LdMT	LdBPK_131590.1	Miltefosine transporter	Phospholipid-translocating ATPase	Miltefosine	*L. donovani*	Partially (Coelho et al., 2012; Mondelaers et al., 2016; Shaw et al., 2016; Srivastava et al., 2017)	Perez-Victoria et al., 2003
	LinJ.34.2310	Phosphatase 2C-like proteins	Catalytic activity	Methotrexate	*L. infantum*	No	Gazanion et al., 2016
	LinJ.34.2320	Phosphatase 2C-like proteins	Catalytic activity	Methotrexate	*L. infantum*	No	Gazanion et al., 2016
	LinJ.06.1010	Leucine Rich Repeat, putative	Protein binding	Pentamidin/ paromomycin	*L. infantum*	No	Gazanion et al., 2016
	LinJ.12.0610	Serine/threonine phosphatase	Hydrolase activity and ion binding	SbIII	*L. infantum*	No	Gazanion et al., 2016
	LinJ30.2270	Phospholipid-translocating ATPase	Transmembrane transporter	Miltefosine	*L. infantum*	No	Gazanion et al., 2016
	LinJ.29.2250	C-8 sterol isomerase	Isomerase	Miltefosine	*L. infantum*	No	Gazanion et al., 2016

* *Strain used for the generation of the cosmid library*;

** biological validation in field isolates

## The framework of “reverse” epidemiology

The examples described above are testimony to the success of cosmid-based, functional screening approaches to discover genetic loci in *Leishmania* that are linked to parasite virulence, tissue tropism, and drug resistance. However, even though these loci may represent potential biomarkers with important prognostic value, there are no dedicated, concerted efforts for their validation in clinically relevant settings. One exception includes CLrP, whose increased abundance on RNA and protein levels were correlated with increased Sb resistance in field isolates, albeit only a small number of isolates were used in these studies (Kumar et al., 2010b; Das et al., 2015). For other loci, clinical validation of the functional screening results can be ambiguous, with for example the MRPA and PTR1 genes of the H-locus having been either strictly, partially, or not correlated to Sb resistance in different epidemiological studies (Decuypere et al., 2005, 2012; Mittal et al., 2007; Mukherjee et al., 2007; Mukhopadhyay et al., 2011). Such divergent results may be explained by the polyclonal structure of parasite field isolates and their geographic adaptation, with different resistance mechanisms being selected in genetically distinct isolates (Decuypere et al., 2012). This possibility is supported by our recent demonstration that genetic mosaicism in an individual *L. donovani* strain can drive polyclonal adaptation, suggesting that different resistance mechanisms may co-exist in sub-populations of any given isolate (Prieto Barja et al., 2017). Such intra-strain specific, polyclonal fitness gain is further supported by the cosmid selection of different genetic loci in response to the same selection pressure applied on a single parasite population *in vitro* or during animal infection (Cotrim et al., 1999; Dahlin-Laborde et al., 2005; Gazanion et al., 2016). Indeed, such clonal phenotypic variability in a given parasite isolate has been recently documented in *L. amazonensis*, with important differences in culture proliferation and pathogenic potential observed in untransfected sub-clones or parasites transfected with individual cosmids selected *in vivo* for increased parasite infectivity (Espiau et al., 2017). Finally, other genes associated with drug resistance or susceptibility identified in cosmid screens failed to be validated in clinical studies such as LdMT, whose mutations were correlated to MIL resistance in promastigotes in culture but could not be associated with MIL resistance or treatment failure in the field (Bhandari et al., 2012). Likewise, PRP1 that has been implicated *in vitro* in resistance to pentamidine with reported cross-resistance to SbIII, did not show increased expression in Sb resistant field isolates (Decuypere et al., 2005, 2012).

**Figure 1:**
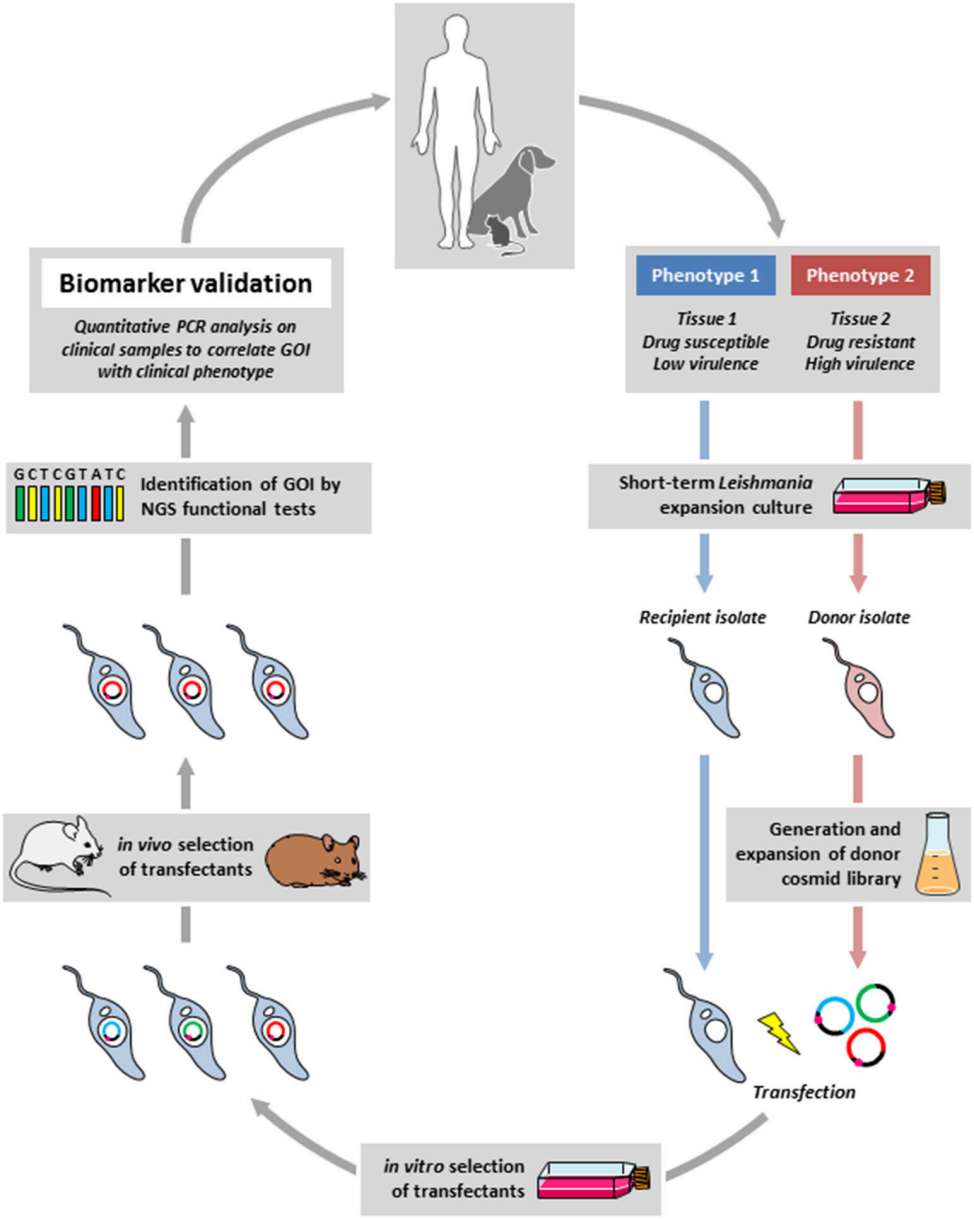
Outline of the reverse epidemiology framework. Field isolates from infected humans or animal reservoirs showing a defined difference in clinical phenotype (e.g., drug susceptibility) will be briefly expanded in culture, a cosmid library will be generated from the donor strain (in red) that shows the phenotype of interest (e.g., drug resistance), which then will be transfected into the recipient strain (in blue) that will be subjected to a gain-of-function screen *in situ* using experimental mouse or hamster infection (in the presence of drug in our example). The selected gene(s) of interested (GOI) will be identified by next generation sequencing (NGS). Correlating the identified genes with the clinical phenotype in dedicated epidemiological studies will then validate the new biomarker.

Drawing from these examples we propose an experimental framework for the discovery of biomarker candidates by combining functional genetic screens in relevant animal models to reveal loci of interest, which then are validated by dedicated clinical and epidemiological investigations (Figure 1). In this approach, a cosmid library is prepared from parasites freshly derived from clinical isolates that show a phenotype of interest (donor strain). The gene(s) that express this phenotype are identified by transfecting a relevant recipient *Leishmania* strain and recovery of cosmids from transfectants that gained the phenotype under investigation. These genes can then be validated as biomarkers by quantitative PCR analysis directly applied on clinical samples. Thus, in contrast to classical biomarker discovery, where epidemiological field studies establish a correlation between a clinical phenotype and a genetic locus that then is validated *in vitro* or in animal studies, the epidemiological protocol we propose is in reverse from lab-based studies back to the field. Even though this approach has its drawbacks (e.g., clinical manifestations caused by gene inactivation or gene deletion cannot be revealed), it provides several interesting advantages that immediately overcome important bottlenecks in *Leishmania* biomarker discovery. First, it is independent of long-term culture that can have an important impact on the parasite genome thus interfering with epidemiologically relevant information. Second, the screening is performed *in situ* in infected animals under environmental constraints that correspond to the clinical setting, thus allowing for the selection of physiologically highly relevant loci. Third, large amounts of parasite can be recovered from different tissues of the infected animals, which can be subjected to direct and even single cell sequencing, thus informing on mechanisms of polyclonal adaptation that may be relevant to the field. Finally, this approach will overcome ethical concerns associated with applying direct genome sequencing on human tissue samples as the cosmid-identified loci will be studied in clinical samples by simple qPCR analysis.

In conclusion, our reverse epidemiology approach exploits genetic amplification for biomarker discovery and thus mimics the very mechanism that has been linked to *Leishmania* genomic adaptation and fitness gain in the field and in culture (Dumetz et al., 2017; Prieto Barja et al., 2017). Cosmid-based functional genetic screening *in situ* linked to clinical validation thus represents a powerful framework that can fill an important gap in the currently rather desolate state of *Leishmania* biomarker discovery, which is challenged by the absence of robust protocols for direct tissue sequencing of parasites in human clinical samples, and the genetic bias caused by parasite long-term culture applied in current epidemiological investigations.

## Author contributions

LP wrote the chapter on the use of cosmid libraries regarding virulence and tropism, PP wrote the chapter on the use of cosmid libraries regarding drug resistance, GS corrected the manuscript and wrote introduction and the last chapter detailing the experimental framework.

### Conflict of interest statement

The authors declare that the research was conducted in the absence of any commercial or financial relationships that could be construed as a potential conflict of interest.
